# Supplementary material for: Distinct and evolutionary conserved structural features of the human nuclear exosome complex
Source: eLife. 2018 Jul 26;7:e38686. doi: 10.7554/eLife.38686 (PMC6072439; doi:10.7554/eLife.38686)
Supplement: Figure 7—source data 1. [file elife-38686-fig7-data1.docx]

| Label | ID | Genotype |
| --- | --- | --- |
| MTR4 | scPR272 | *MAT***a** {MTR4} |
| *mtr4*∆::kanMX4 | scPR275 | *MAT***a** {W303, *mtr4*Δ::kanMX4}[YCplac33-MTR4] |
| MTR4*-EGFP* | scPR305 | *MAT***a** {W303, *mtr4*-*EGFP*::kanMX4}[YCplac33-MTR4] |
| *mtr4*-R887E-R890E-R891E-*EGFP* | scPR320 | *MAT***a** {W303, *mtr4*-R887E-R890E-R891E-*EGFP*::kanMX4}[YCplac33-MTR4} |
| *mtr4*-∆SK-*EGFP* | scPR322 | *MAT***a** {W303, *mtr4*-ΔSK-*EGFP*::kanMX4} [YCplac33-MTR4] |
